# Testing Danegaptide Effects on Kidney Function after Ischemia/Reperfusion Injury in a New Porcine Two Week Model

**DOI:** 10.1371/journal.pone.0164109

**Published:** 2016-10-19

**Authors:** Chris Amdisen, Anna K. Keller, Rie Schultz Hansen, Rikke Nørregaard, Søren Palmelund Krag, Ulla Møldrup, Michael Pedersen, Bente Jespersen, Henrik Birn

**Affiliations:** 1 Institute of Clinical Medicine, Aarhus University, Aarhus, Denmark; 2 Department of Renal Medicine. Aarhus University Hospital, Aarhus, Denmark; 3 Department of Urology, Aarhus University Hospital, Aarhus, Denmark; 4 Zealand Pharma A/S, Glostrup, Denmark; 5 Department of Pathology, Aarhus University Hospital, Aarhus, Denmark; 6 Comparative Medicines Lab, Aarhus University, Aarhus, Denmark; 7 Department of Biomedicine, Aarhus University, Aarhus, Denmark; University of Sao Paulo Medical School, BRAZIL

## Abstract

**Introduction:**

Ischemia/reperfusion injury (I/R-I) is a leading cause of acute kidney injury (AKI) and is associated with increased mortality. Danegaptide is a selective modifier of the gap junction protein connexion 43. It has cytoprotective as well as anti-arrhythmic properties and has been shown to reduce the size of myocardial infarct in pigs. The aim of this study was to investigate the ischemia-protective effect of Danegaptide in a porcine renal I/R-I model with two weeks follow up.

**Methods:**

Unilateral renal I/R-I was induced in pigs by clamping the left renal artery over a two hour period. The model allowed examination of renal blood flow by magnetic resonance imaging (MRI) and the measurement of single kidney GFR two weeks after injury. Eleven animals were randomized to Danegaptide-infusion while nine animals received placebo. Kidney histology and urinary neutrophil gelatinase-associated lipocalin (NGAL) excretion were included as markers of AKI.

**Results:**

Unilateral kidney I/R-I resulted in an immediate ~50% GFR reduction, associated with a four-fold increase in urinary NGAL-excretion. Fourteen days after I/R-I, the total GFR was ~75% of baseline with a significantly lower GFR in the injured left kidney compared to the right kidney. No differences in GFR were observed between the treated and non-treated animals immediately after I/R-I or at Day 14. Furthermore, no differences were observed in the urinary excretion of NGAL, renal blood flow or other markers of renal function.

**Conclusions:**

As expected this porcine renal I/R-I model was associated with reduced GFR two weeks after injury. Danegaptide did not improve renal function after I/R-I.

## Introduction

Ischemia Reperfusion Injury (I/R-I) is the one of the leading causes of acute kidney injury (AKI) in critically ill patients [1]. Renal I/R-I is seen in one of its most pure forms after aortic surgery, with clamping of the renal artery leading to warm ischemia. In this setting AKI is associated with an increased risk of septic shock and increased long-term mortality [2,3]. In renal transplantation after deceased brain death donation, delayed graft function after I/R-I is also associated with a poorer graft outcome [4,5].

Several porcine models have been established to study I/R-I and different strategies to attenuate the deleterious effects of such I/R-I have been considered. These studies examines either the acute effects [6–8] or the long term effects of I/R-I and possible interventions [9–11]. Furthermore, I/R-I is induced by a variety of methods, including clamping of renal vessels [6], intra-arterial ballooning of the aorta [12], or renal autotransplantation [13]. Some of the studies on attenuation of AKI have produced promising results, but the results have not been conclusive [6,10]. This is probably partly related to lack of a perfect animal model for preclinical trials.

Several pharmacological strategies have been applied to protect against or attenuate renal I/R-I, but so far none of these have proven effective in humans [6–8,14]. Both Danegaptide, a dipeptide, and its analogue Rotigaptide, were originally developed as antiarrhythmic agents. However studies in dogs and pigs have shown that they also mediate cytoprotective effects during myocardial I/R-I [15,16], most likely via the gap junctional hemichannel connexin 43 [17]. In the cardiomyocyte mitochondria, connexin 43 plays a significant role in the development of ischemic injury as well as cardioprotection by ischemic preconditioning. It has been shown that opening of mitochondrial connexin 43 channels prior to ischemia or reperfusion provides protection against I/R-I [18–20]. Connexin 43 has been identified in all segments of the kidney, including the gap junctions between the podocytes [21–24], in the renal vasculature [25] as well as in renal tubular cells [16,26], [27]. In the kidney, formation of Cx43 mediated gap junctions allows for intercellular communication by formation of hemichannels, that facilitate cellular secretion of large signaling molecules and mediate calcium signaling and vascular conduction [28]. ATP depletion in primary cultures of human proximal tubule cells has been related to activation of hemichannels with properties of connexin 43 [29], and upregulation of connexin 43 has been associated with protection against renal I/R-I mediated by the glycogen synthase kinase 3-inhibitor TDZD-8 [30]. This suggests that modulation of connexin 43 function by Danegaptide may provide protective effects in relation to renal I/R-I.

The aims of this study were firstly to establish a porcine unilateral kidney I/R-I model, and secondly to examine the potential renal protective effects of intravenous administration of Danegaptide during ischemia as assessed by total and single kidney glomerular filtration rate (GFR), tubular injury marker, and kidney histology, as well as renal blood flow and renal deoxygenation.

## Materials and Methods

### Animal handling and anaesthesia

This study was approved by the Danish Animal Experiments Inspectorate (no. 2013-15-2934-00925). Female Landrace pigs weighing mean 42 kg (n = 24) were fasted overnight with free access to water. Anaesthesia was induced using a bolus of intravenous propofol (3–4 mg/kg) followed by continuous infusion of propofol (3 mg/kg/h) and fentanyl (12.5 mg/h/kg). Mechanical ventilation was maintained with 40% oxygen and a tidal volume of 10 mL/kg. Expiratory CO_2_ was maintained at a level of 4.5–5.5 kPa. Benzylpenicilin procain (Penovet^®^, 15.000 IE/kg) was administered intramuscularly as an antibiotic prophylaxis before the surgical procedure. The femoral artery was catheterized for blood pressure monitoring and blood sampling. The animals received a bolus of 2 ltrs of normal saline upon arrival followed by a continuous infusion of isotonic Ringer acetate (12.5 mL/kg/h).

### Experimental procedures day 1

**Fig 1:**
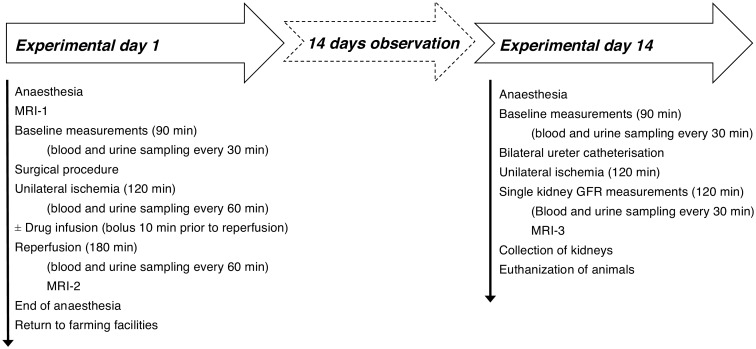
Timeline of experiment.

A timeline of the experiment is provided in Fig 1. Upon anaesthesia baseline blood and urine samples were collected at 30 minute intervals and a bolus and infusion of ^51^Cr-ethylenediaminetetraacetic (EDTA) were initiated to measure urinary ^51^Cr-EDTA clearance.

The left kidney was exposed retroperitoneally under sterile conditions with an incision in the left flank. The renal artery was dissected until the aorta and left kidney I/R-I was induced by clamping the left renal artery using an atraumatic arterial clamp, which was removed after two hours. A change in the kidney color from normal red to grey was observed, indicating ischemia, while normalization of kidney color was observed following removal of the clamp, indicating successful reperfusion. The incision was then closed in two layers. The animals were observed under anaesthesia during the first three hours of reperfusion. Ten minutes prior to reperfusion, the animals received a bolus of Danegaptide or vehicle, followed by continuous infusion for three hours. Following the reperfusion period, the animals were allowed to wake up and returned to the housing facilities for further recovery. During ischemia and reperfusion, blood and urine was collected at 60 min intervals.

### Experimental procedures day 14

The baseline periods, prior to surgery, were similar on the two experimental days (Fig 1). Following baseline sampling on experimental Day 14 both ureters were exposed through a midline incision and catheterized for single kidney urine collection. The catheters where tunneled to the surface through the abdominal wall and the incision was closed. This was followed by two hours of observation, including blood and single kidney urine sampling every 30 min. Following the last sampling on experimental Day 14, the animals were euthanized with a lethal dose of pentobarbital (80 mg/kg) whilst still under anaesthesia.

### Endpoints

The primary endpoint was the GFR of the left, ischemic kidney measured by urinary ^51^Cr-EDTA clearance two weeks after injury. Secondary endpoints included: changes in total GFR after I/R-I; renal plasma perfusion measured by MRI; urinary neutrophil gelatinase-associated lipocalin (NGAL) excretion; and histological evidence of AKI, in addition as any adverse effects from Danegaptide observed during the study including hemodynamic instability measured by blood pressure and heart rate.

### Glomerular filtration rate

GFR was measured as urinary ^51^Cr-EDTA clearance. A bolus of ^51^Cr-EDTA was followed by continuous intravenous infusion. Blood and urine samples were collected at regular intervals as described above and counted in a gamma counter (Perkin Elmer 2480, Automatic Gamma Counter, Wizzard). Correction for background and decay was performed.

### Magnetic resonance imaging

MRI was performed with a 3 T clinical system (Magnetom Avanto; Siemens Healthcare, Erlingen, Germany) as previously described [31,32]. The heart rate was continuously monitored by electrocardiography. For all sequences, we used the accelerated parallel GRAPPA approach. Orthogonal slices on the left and right renal arteries were identified, and measurements of left- and right-side arterial RBF were performed with a 2D phase-contrast gradient-echo sequence. Blood-oxygen-level-dependent (BOLD) MRI was performed on each kidney with a multi-echo gradient-echo sequence [31]. The velocity profiles of the left and right renal arteries were measured from the acquired phase velocity-sensitive images and arterial RBF was determined in units of ml/min by multiplication with the renal artery cross sectional area. Finally, a diffusion-weighted sequence was employed to calculate the apparent diffusion coefficient (ADC), which is a physical parameter sensitive to tissue oedema. The quantitative analyses were performed blinded to the interventions using the Mistar analysis software (Apollo Medical Imaging Technology, Melbourne, Australia), and all analyses were performed for both kidneys and for cortical and medullary regions [32].

### NGAL

NGAL concentrations were measured using a NGAL ELISA kit (Bioporto, Hellerup, Denmark) according to the manufacturer’s instruction. Urinary NGAL excretion rates were calculated by multiplying with urinary output.

### Histology

Tissue samples from the kidneys were collected at the end of the experiment. The samples were cut and immersion fixed in 4% formalin immediately after the kidneys were collected. They were then stored in formalin for 24hrs followed by 0.1 M phosphate buffered saline until dehydrated, embedded in paraffin, and sectioned using a standard microtome (Microm 355; Thermo Fisher Scientific, Waltham, Maine, USA). The sections were stained by hematoxylin and eosin and evaluated by an experienced pathologist blinded to the intervention. Histological evidences of kidney injury were graded from 0–4 based on the following parameters: tubular injury, tubular casts, interstitial inflammation, glomerular damage, and vacuolization of the cytoplasma.

### Preparation of Danegaptide (ZP1609)

ZP1609 ((2S,4R)-1-(2-aminoacetyl)-4-benzamidopyrrolidine- 2-carboxylic acid) was synthesized at Zealand Pharma (Glostrup, Denmark) and was dissolved in sterile saline. Saline served as vehicle control. The dose for the initial injection (75 μg/kg body weight) was further diluted to 10 ml and slowly given over one minute intravenously and followed by a continuous infusion (57 μg/kg/min iv). The plasma concentration of ZP1609 was analyzed by protein precipitation and HPLC-MS/MS.

### Plasma Danegaptide concentration

Plasma Danegaptide concentrations were measured in all animals by Zealand Pharma.

The plasma concentration was analyzed by protein precipitation and HPLC-MS/MS

### Statistics

Data are presented as means ±95% confidence intervals. Student’s T-test was used to compare means. All statistics were performed using GraphPad Prism version 5.0 for Mac OS X.

## Results

### Induction of unilateral kidney I/R-I in pigs

Four animals were excluded from the study, one due to faulty catheterization of the bladder, one due to hypoxia at the end of the anaesthesia, while two animals were lost due to a technical defect in the mechanical ventilator. The twenty included animals had a mean weight of 42.2 kg (41.1–43.3 kg) and 45.2 kg (44.1–46.3 kg) on experimental Day 1 and 14, respectively.

**Fig 2:**
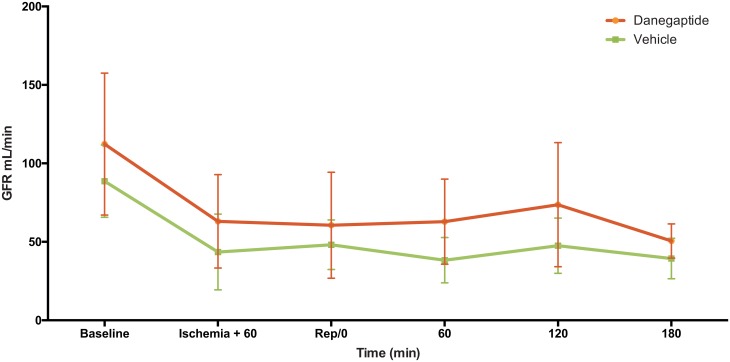
**GFR day 1-** GFR measured by ^51^Cr-EDTA clearance. Error bars represents 95% CI.

All GFR measurements on Day 1 represent total GFR, including both the left and right kidneys (Fig 2). The mean baseline GFR of vehicle treated animals was 89 mL/min (69–109 mL/min). After clamping of the left renal artery total GFR was significantly reduced to 49% of baseline, this remained unchanged during reperfusion.

**Fig 3:**
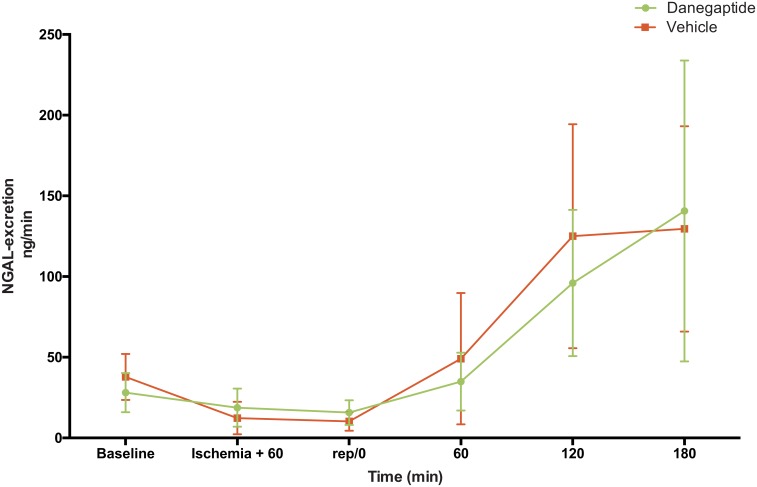
**NGAL-excretion day 1-** NGAL excretion rate in urine. Error bars represents 95% CI.

Following I/R-I in vehicle treated animals, an approx. four-fold increase in urinary NGAL excretion rate was identified. This increase in urinary NGAL was observed one hour after reperfusion reaching a plateau after two hours. (Fig 3).

**Fig 4:**
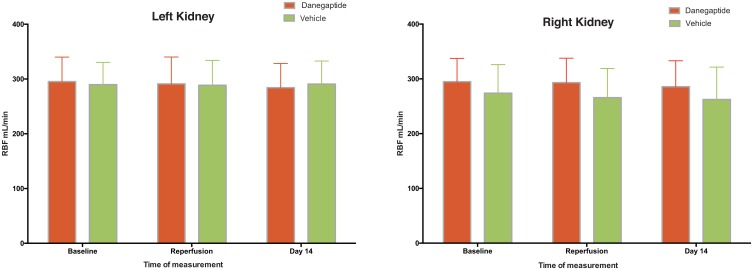
**Renal Blood Flow—**RBF measured by MRI at baseline, after reperfusion and 14 days after I/R-I. Error bars represents 95% CI.

No significant differences or changes were observed in the renal blood flow of the left nor the right kidney when comparing before and after I/R-I (Fig 4). The oxygenation (BOLD) and ADC values are shown in Table 1, and these parameters showed no changes following I/R-I.

**Table 1:** Tissue metabolic activity (BOLD) and edema (ADC) measured by MRI in the left and right kidneys from Danegaptide (D) and vehicle (V) treated animals on experimental Day 1 (baseline and after reperfusion) and Day 14, respectively.

	Bold cortex	Bold Medulla	ADC (cortex)	ADC (Medulla)
**Baseline**				
Left (D)	12,4	30,7	2,1	1,7
Left (V)	12,2	31,4	2,0	1,6
Right (D)	12,1	31,4	2,0	1,7
Left (V)	12,1	31,2	2,0	1,6
**Reperfusion**				
Left (D)	12,9	31,8	2,1	1,7
Left (V)	12,4	30,9	2,1	1,6
Right (D)	12,9	31,9	2,1	1,7
Left (V)	12,5	31,1	2,0	1,6
**Day 14**				
Left (D)	13,4	31,9	2,1	1,7
Left (V)	13,3	31,8	2,1	1,7
Right (D)	13,3	31,4	2,1	1,7
Left (V)	13,3	31,5	2,1	1,8

### Renal function at two weeks after I/R-I

**Fig 5:**
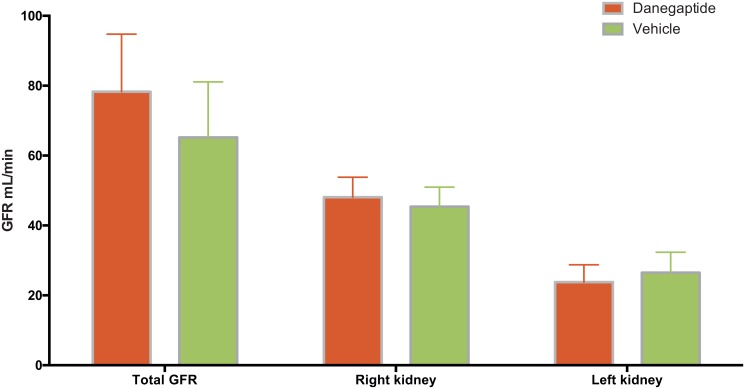
**GFR day 2—**GFR measured by ^51^CR-EDTA clearance. Error bars represents 95% CI.

On experimental Day 14, both total (prior to catherization of the ureters) and single kidney GFR was measured (Fig 5). Total GFR was 65 mL/min (51–79 mL/min), representing 74% of baseline at day 1. However, this decline was not statistically significant. GFR of the injured left kidney in vehicle-treated animals was significantly smaller compared to the right kidney (58% of the right kidney GFR). Histological evidence of low degree inflammation and vacuolization was identified in the vehicle treated and injured left kidneys at Day 14. However, this was not significantly different from the morphology observed in the right kidney, which had not been clamped (Table 2). This is consistent with a mild stage of ischemia induced tubular necrosis. No changes in renal blood flow, BOLD or ADC were observed fourteen days after I/R-I (Fig 4 and Table 1).

**Table 2:** Histological grades. The numbers are expressed as a mean for all animals in the group. In the top table, the Danegaptide treated and the vehicle animals are compared. In the bottom table, the left, injured kidney is compared to the right, contralateral kidney.

	**Tubular injury**	**Tubular casts**	**Inflammation**	**Glomerular damage**	**Vacuolization**
Left (D)	0	0	0,82	0	2,64
Left (D)	0	0	0,56	0	2,67
P-value			0,48		0,89
Right (D)	0,1	0	0,1	0	2,3
Right (V)	0	0	0,22	0	1,89
P-value	0,36		0,61		0,18
	**Tubular injury**	**Tubular casts**	**Inflammation**	**Glomerular damage**	**Vacuolization**
Left (D)	0	0	0,82	0	2,64
Left (D)	0,1	0	0,1	0	2,3
P-value	0,31		0,02		0,14
Right (D)	0	0	0,56	0	2,67
Right (V)	0	0	0,22	0	1,89
P-value			0,33		0,02

### Danegaptide administered during ischemic injury does not protect against renal I/R-I

The Danegaptide treated animals revealed a mean baseline GFR of 112 mL/min (72–152 mL/min) on experimental Day 1 (Fig 1), which despite randomization was 20% higher than in the vehicle treated animals. This difference was not significant (P = 0.34) and remained throughout experimental day 1 with similar, relative changes in GFR on Day 1 when comparing the two groups. There were no differences between the two experimental groups on experimental Day 1 or Day 14 with respect to weight, body temperature, arterial blood pH, P-sodium, P-potassium, P-glucose, or P-lactate (data not shown).

On experimental Day 14, total GFR (Fig 5) was decreased to 78 mL/min (64–92 mL/min) which still tended to be higher than in the vehicle-treated animals, (p = 0.13) representing a similar, relative reduction when compared to baseline (70% of baseline in Danegaptide treated animals). There was no difference in left kidney GFR between the Danegaptide-treated and the vehicle-treated animals (27 mL/min (22–32 mL/min) versus 24 mL/min (20–28 mL/min), respectively, P = 0.25). Histological examination revealed no differences between the two experimental groups.

The renal blood flow, BOLD or ADC (measured by MRI) did not change significantly throughout the experimental period in the Danegaptide treated animals, nor did they differ from the vehicle animals (Fig 4 and Table 1). Furthermore, no significant differences were observed in any of the parameters when comparing the Danegaptide-treated and the vehicle-treated animals (Fig 4. and Table 1).

**Fig 6:**
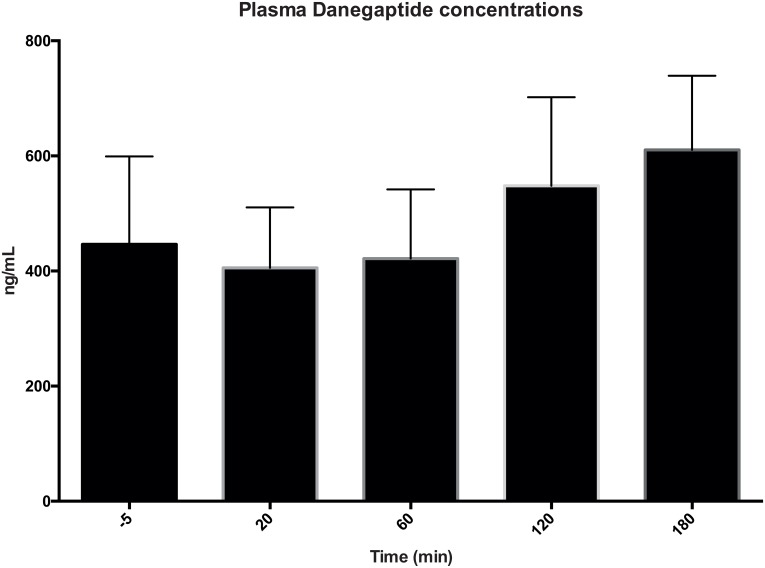
**Plasma Danegaptide concentration:** Measured on day 1. Error bars represents 95% CI.

An appropriate increase in the plasma concentration of Danegaptide was observed during the infusion and reperfusion period (Fig 6). Danegaptide was well tolerated without adverse effects on animal behaviour or surgical complications. The mean arterial pressure and heart rate were stable throughout the experiment period on both Day 1 and Day 14, and these parameters were similar in the Danegaptide-treated and vehicle-treated animals (data not shown).

## Discussion

We have successfully established a model of unilateral renal I/R-I as evaluated by measuring GFR with an increase in urinary NGAL excretion from 60 min after reperfusion. The decrease in left kidney GFR at Day 14, with no total loss of kidney function, is consistent with persistent damage two weeks after ischemia-reperfusion injury, indicating that the model is appropriate for studying this later outcome of potential, protective interventions at the time of injury. The experimental model proved to be reproducible and manageable allowing for trial interventions. It is based on a preliminary study in pigs comparing the effects of one or two hours of warm ischemia times (unpublished data). This study showed that the significant reduction in single kidney GFR following one hour of warm ischemia was reversible within a few hours of reperfusion, which was not the case after two hours of warm ischemia time. These observations are supported by other studies [6,10,33]. In contrast to previous observations in a porcine renal-transplantation model [34,35] no changes in renal blood flow was observed following I/R-I, neither in the cortex nor the medulla. Also, no difference in blood flow was observed between the left injured kidney and the right kidney.

The study did not include a sham operated group and thus, does not allow us to compare renal function and biomarkers with baseline data. A further limitation of the experimental model is its inability to measure single kidney GFR on the first experimental day, and thus, to measure the acute, functional changes in the injured left kidney after I/R-I. The surgical procedure was designed to be as minimally invasive as possible, and ureters were not catheterized on the first experimental day. Thus, urine was collected from both kidneys on Day 1 through the bladder, which may explain the greater variation in GFR on Day 1 compared to Day 14. The variation in GFR was reduced following catheterization of the ureters on Day 14, which is essential for the evaluation of the primary endpoint of this study. Other studies avoided this issue by using a single-kidney model [10,11]. Single-kidney nephrectomy allows for measuring the function of the remaining, damaged kidney using total urinary output. However, this approach has been associated with a greater loss of animals dying during recovery even with shorter renal ischemia times than in this study. In studies by Jayle et al. four out of eight animals died after 90 minutes of warm ischemia while two out of eight and two out of seven animals died after 60 minutes [9,36]. In a study by Abreu et al., five of fifteen animals died after 120 minutes of warm ischemia. We did not nephrectomize our animals, in order to minimize the surgical stress response, to minimize loss of animals, and to avoid the effect of nephrectomy on the remaining kidney, so as not to confound our results.

We evaluated the potential renal protective effects of Danegaptide on renal I/R-I, showing no acute or persistent, conditioning effect as evaluated by; measured GFR, NGAL excretion rate, or by the observed histological parameters. In addition this was consistent with no difference in renal blood flow, BOLD or ADC, being observed between the Danegaptide-treated and the vehicle-treated animals. On the first experimental day, GFR of the Danegaptide-treated animals was insignificantly higher than the vehicle treated animals. This difference remained throughout the experiment, despite randomization. We did not investigate the changes in expression and function of connexin 43 after the I/R-I; however, as no effect of Danegaptide was identified, such studies may currently be of limited clinical interest.

Infusion of Danegaptide was well tolerated and did not cause any adverse effects in pigs with AKI. In particular, we observed no differences in hemodynamics, animal behaviour, or surgical complications between Danegaptide and vehicle treated pigs when observed for two weeks after the infusion and I/R-I. Thus, the applied dose of Danegaptide appeared to be well tolerated in pigs with AKI. The protocol for infusion and dosing of Danegaptide was based on a previous study by Skyschally et al. using Göttingen minipigs weighing 20–40 kilos, demonstrating the cardioprotective effects of Danegaptide [15] The concentrations of Danegaptide measured in our study (data not shown) were almost twice the concentrations in this reference study [15].

It has been hypothesised that the effect of Danegaptide is dependent on the initiation of its cellular effects prior to reperfusion. If administered after arterial occlusion the exposure of the ischemic cells to Danegaptide will depend on collateral blood supply as observed e.g. in the infarct marginal zone of the myocardium following occlusion of one coronary artery. Since the pig kidney is supplied by end-arteries with essentially no branching vessels, it is likely that the drug did not reach the ischemic renal tissue before reperfusion. Thus, our study cannot exclude a possible effect of Danegaptide if administered prior to I/R-I or by a method allowing for exposure during ischemia, e.g. by machine perfusion of a kidney transplant prior to engraftment. As the primary aim of this study was to investigate the potential, beneficial effects of Danageptide two weeks after reperfusion, possible, clinically important, early effects cannot be excluded. This would require reliable functional and histological evaluation at earlier timepoints after I/R-I. Also, this study was performed in juvenile pigs with greater regenerative capabilities possibly reducing the potential, additive protective effect of Danegaptide. A previously demonstrated protective effect in hearts was observed in adult, Gøttingen pigs [15].

In conclusion, we have successfully established a model of unilateral renal I/R-I in pigs with a 14 day observation period, allowing the study of I/R-I effects on renal function, damage, and blood flow. Evidence of AKI was identified as a persistent decrease in GFR at 14 days and the early increased urinary excretion of NGAL. Using this model, we observed no renoprotective effects of Danegaptide when infused during and after occlusion of the renal artery. Infusion of Danegaptide in pigs with AKI was well tolerated without adverse effects.

## Supporting Information

S1 File (ZIP)
