# Supplementary material for: Testing Danegaptide Effects on Kidney Function after Ischemia/Reperfusion Injury in a New Porcine Two Week Model
Source: PLoS One. 2016 Oct 19;11(10):e0164109. doi: 10.1371/journal.pone.0164109 (PMC5070773; doi:10.1371/journal.pone.0164109)
Supplement: S1 File — (ZIP) [file pone.0164109.s001.zip › PLOS-one/Histologi/Histo tabel.pdf]

	**Tubular injury**	**Tubular casts**	**Inflammation**	**Glomerular damage**	**Vacuolization**
Left (D)	0	0	0,82	0	2,64
Left (V)	0	0	0,56	0	2,67
P-value			*0,48*		*0,89*
Right (D)	0,1	0	0,1	0	2,3
Right (V)	0	0	0,22	0	1,89
P-value	*0,36*		*0,61*		*0,18*
