# Supplementary material for: Testing Danegaptide Effects on Kidney Function after Ischemia/Reperfusion Injury in a New Porcine Two Week Model
Source: PLoS One. 2016 Oct 19;11(10):e0164109. doi: 10.1371/journal.pone.0164109 (PMC5070773; doi:10.1371/journal.pone.0164109)
Supplement: S1 File — (ZIP) [file pone.0164109.s001.zip › PLOS-one/Histologi/tabel.docx]

|  | **Tubular**  **injury** | **Tubular**  **casts** | **Inflammation** | **Glomerular**  **damage** | **Vacuolization** |
| --- | --- | --- | --- | --- | --- |
| Left (D) | 0 | 0 | 0,82 | 0 | 2,64 |
| Left (V) | 0 | 0 | 0,56 | 0 | 2,67 |
| *P-value* |  |  | *0,48* |  | *0,89* |
|  |  |  |  |  |  |
| Right (D) | 0,1 | 0 | 0,1 | 0 | 2,3 |
| Right (V) | 0 | 0 | 0,22 | 0 | 1,89 |
| *P-value* | *0,36* |  | *0,61* |  | *0,18* |
