# Supplementary material for: Testing Danegaptide Effects on Kidney Function after Ischemia/Reperfusion Injury in a New Porcine Two Week Model
Source: PLoS One. 2016 Oct 19;11(10):e0164109. doi: 10.1371/journal.pone.0164109 (PMC5070773; doi:10.1371/journal.pone.0164109)
Supplement: S1 File — (ZIP) [file pone.0164109.s001.zip › PLOS-one/Z1/Frysepr├╕ver Z1 B1 14.01.14.doc]

**14.01.14**

Prøver Z1 B1

| Blod | TBARS | NGAL | Væsketal |  |  |  |
| --- | --- | --- | --- | --- | --- | --- |
| T: Basel | 2 | 2 | 1 |  |  |  |
| Fi T:0 | 1 | 1 | 1 |  |  |  |
| Fi T:30 | 1 | 1 | 1 |  |  |  |
| Fi T:60 | 1 | 1 | 1 |  |  |  |
| Fi T:90 | 1 | 1 | 1 |  |  |  |
| T:0 | 1 | 1 | 1 |  |  |  |
| T:60 | 1 | 1 | 1 |  |  |  |
| T:120 | 1 | 1 | 1 |  |  |  |
| T:180 | 1 | 1 | 1 |  |  |  |
| T:240 | 1 | 1 | 1 |  |  |  |
| T:300 | 1 | 1 | 1 |  |  |  |
|  |  |  |  |  |  |  |
| Urin | L-FABP | NAG | NGAL | TBARS | Væsketal | Ekstra |
| T: Basel | 1 | 1 | 1 | 1 | 1 | 2 |
| Fi T:30 | 1 | 1 | 1 | 1 | 1 | 2 |
| Fi T:60 | - | - | - | - | - | - |
| Fi T:90 | 1 | 1 | 1 | 1 | 1 | 2 |
| T:60 | 1 | 1 | 1 | 1 | 1 | 2 |
| T:120 | 1 | 1 | 1 | 1 | 1 | - |
| T:180 | 1 | 1 | 1 | 1 | 1 | 2 |
| T:240 | 1 | 1 | 1 | 1 | 1 | 2 |
| T:300 | 1 | 1 | 1 | 1 | 1 | 2 |
