# Supplementary material for: Testing Danegaptide Effects on Kidney Function after Ischemia/Reperfusion Injury in a New Porcine Two Week Model
Source: PLoS One. 2016 Oct 19;11(10):e0164109. doi: 10.1371/journal.pone.0164109 (PMC5070773; doi:10.1371/journal.pone.0164109)
Supplement: S1 File — (ZIP) [file pone.0164109.s001.zip › PLOS-one/Z1/Z1 B1.doc]

**Gris Z1 B1**

Fi T:0 1 time efter ^51^ Cr bolus.

Analyser heparin-plasma: Crea, Carbamid, K, Na, NGAL og TBARS

Analyser Urin: Crea, Carbamid, K, Na, L-FABP, NAG, NGAL og TBARS

Dato: 14.01.14

| Tid i min |  | Heparin  Plasma  + EDTA  Plasma | Urin  Tid  Kl: | Urin  Volumen  ml |
| --- | --- | --- | --- | --- |
| Baseline  Blod + urin | Kl. 08:55 | X | Urin Kl. 09:00  X |  |
| Bolus ^51^ Cr  **MR** | Kl. 09:00 |  |  |  |
| Fi T:0  Veneblod | Kl. 10:26 | X | Start opsamling  Kl. 10:27 |  |
| Fi T:30 | Kl. 11:02 | X | Kl. 11:04 | ml. 5 |
| Fi T:60 | Kl. 10:27 | X | Kl. 10:27 | ml. 6 |
| Fi T:90 | Kl. 11:52 | X | Kl. 11:52 | ml. 6 |
| Iskæmi Start  T:0 | Kl. 12:30 | X | Start opsamling  Kl. 12:30 |  |
| T:60 | Kl. 13:30 | X | Kl. 13:30 | **GFR 0,5 ml**  ml. 0,5 |
| Bolus Danegapeptid 10 min før Reperfution | Kl. 14:20 |  |  |  |
| Iskæmi slut Reperfution  T:120 | Kl. 14:30 | X | Kl. 14:30 | ml 4 |
| T:180 | Kl. 15:30 | X | Kl. 15.30 | ml 107 |
| T: 240  **MR** | Kl. 16:30 | X | Kl. 16:30 | ml. 99 |
| T:300 | Kl. 17:30 | X | Kl. 17.30 | ml. 265 |

Plasma til GFR: 3 ml	Urin til GFR: 1 ml
