# Supplementary material for: Testing Danegaptide Effects on Kidney Function after Ischemia/Reperfusion Injury in a New Porcine Two Week Model
Source: PLoS One. 2016 Oct 19;11(10):e0164109. doi: 10.1371/journal.pone.0164109 (PMC5070773; doi:10.1371/journal.pone.0164109)
Supplement: S1 File — (ZIP) [file pone.0164109.s001.zip › PLOS-one/Z1/Z1 B2.doc]

**Gris Z1 B2**

T:0 H+V 1 time efter ^51^ Cr bolus

Analyser heparin-plasma: Crea, Carbamid, K, Na, NGAL og TBARS

Analyser Urin: Crea, Carbamid, K, Na, L-FABP, NAG, NGAL og TBARS

Dato: 28.01.14	Vægt: 47,8 kg

| Tid i min | Kl. | Heparin  Plasma  + EDTA  Plasma | Urin  Højre  Tid  Kl: | Urin  Højre  Volumen  ml | Urin  Venstre  Tid  Kl: | Urin  Venstre  Volumen  ml |
| --- | --- | --- | --- | --- | --- | --- |
| Baseline  Blod + urin H+V | Kl. 10:35 | X | Urin  Kl. 10:35 |  |  |  |
| Bolus ^51^ Cr | Kl. 10:40 |  |  |  |  |  |
| T:0  H+V | Kl. 11:40 | X | H+V  Start opsamling: 11:40 |  |  |  |
| T:30  H+V | Kl. 12:10 | X | H+V  Kl: 12:10 | H+V  **ml: 0,2** | **GFR**  **200 µl** | Forkert placering af kat. |
| T:60  H+V | Kl. 12:40 | X | H+V  Kl: 12:40 | H+V  **ml: 0** | **- GFR** | Fyldt blære ca 300 ml |
| T:90  H+V | Kl. 13:10 | X | H+V  Kl: 13:10 | H+V  **ml: 0** | **- GFR** |  |
| Katedre indsættes |  |  |  |  |  |  |
| T:0 | Kl. 14:00 | X | Start opsamling  Kl. 14:00 |  | Start opsamling  Kl.14:00 |  |
| T:30 | Kl. 14:30 | X | Kl: 14:30 | **ml: 14** | Kl: 14:30 | **ml: 40** |
| T:60 | Kl. 15:00 | X | Kl: 15:00 | **ml: 13** | Kl: 15:00 | **ml: 33** |
| T:90 | Kl. 15:34 | X | Kl: 15.34 | **ml: 17,5** | Kl: 15:34 | **ml: 42** |
| T:120  MR | Kl. 16:00 | X | Kl: 16:00 | **ml: 12** | Kl: 16:00 | **ml: 39** |

Højre Nyre vægt: 114 g	Plasma til GFR: 3 ml	**Foto af delt nyre**

Venstre nyre vægt: 124 g	Urin til GFR: 1 ml
