# Supplementary material for: Testing Danegaptide Effects on Kidney Function after Ischemia/Reperfusion Injury in a New Porcine Two Week Model
Source: PLoS One. 2016 Oct 19;11(10):e0164109. doi: 10.1371/journal.pone.0164109 (PMC5070773; doi:10.1371/journal.pone.0164109)
Supplement: S1 File — (ZIP) [file pone.0164109.s001.zip › PLOS-one/Z10/Z10 B2.doc]

**Gris Z10 B2	Dato: 25.03.14/IR**

T:0 H+V 1 time efter ^51^ Cr bolus

Analyser heparin-plasma: Crea, Carbamid, K, Na, NGAL og TBARS

Analyser Urin: Crea, Carbamid, K, Na, L-FABP, NAG, NGAL og TBARS

| Tid i min |  | Heparin  Plasma  + EDTA  Plasma | Urin  Højre  Tid  Kl: | Urin  Højre  Volumen  ml | Urin  Venstre  Tid  Kl: | Urin  Venstre  Volumen  ml |
| --- | --- | --- | --- | --- | --- | --- |
| Baseline  Blod + urin H+V | Kl. 10:30 | + Cystatin C  X | Urin  Kl. 10:35 |  |  | 3 tubes urin til Danny |
| Bolus ^51^ Cr | Kl. 10:33 |  |  |  |  |  |
| T:0  H+V | Kl. 11:31 | X | H+V  Start ops.  11:30 |  |  |  |
| T:30  H+V | Kl. 12:01 | X | H+V  Kl: 12:00 | H+V  **ml: 40** |  |  |
| T:60  H+V | Kl. 12:31 | X | H+V  Kl: 12:30 | H+V  **ml: 120** |  |  |
| T:90  H+V | Kl. 13:01 | X | H+V  Kl: 13:00 | H+V  **ml: 146** |  |  |
| Katedre indsættes |  |  |  |  |  |  |
| T:0 | Kl. 13:51 | X | Start opsamling  Kl. 13:50 |  | Start opsamling  Kl. 13:50 |  |
| T:30 | Kl. 14:21 | X | Kl: 14:20 | **ml: 30** | Kl: 14:20 | **ml: 86** |
| T:60 | Kl. 14:53 | X | Kl: 14:49 | **ml: 23,5** | Kl: 14:49 | **ml: 47** |
| T:90 | Kl. 15:26 | X | Kl: 15:23 | **ml: 42** | Kl: 15:23 | **ml: 92** |
| T:120  MR | Kl. 15:54 | X | Kl: 15:50 | 3 tubes urin til Danny  **ml: 35** | Kl: 15:50 | 3 tubes urin til Danny  **ml: 81** |

Højre Nyre vægt: 118 g	Plasma til GFR: 3 ml	**Foto af delt nyre**

Venstre nyre vægt: 162 g	Urin til GFR: 1 ml
