# Supplementary material for: Testing Danegaptide Effects on Kidney Function after Ischemia/Reperfusion Injury in a New Porcine Two Week Model
Source: PLoS One. 2016 Oct 19;11(10):e0164109. doi: 10.1371/journal.pone.0164109 (PMC5070773; doi:10.1371/journal.pone.0164109)
Supplement: S1 File — (ZIP) [file pone.0164109.s001.zip › PLOS-one/Z13/Z13 B1 med ZP1609.doc]

**Gris Z13 B1**	Dato: 18.03.14/IR

Fi T:0 1 time efter ^51^ Cr bolus.

ZP1609	T:115, T:140, T:180, T:240 og T:300 lige før infutionsstop. 1 ml EDTA-P

Analyser heparin-plasma: Crea, Carbamid, K, Na, NGAL, Cystatin C og TBARS

Analyser Urin: Crea, Carbamid, K, Na, L-FABP, NAG, NGAL og TBARS

| Tid i min |  | Heparin  Plasma  + EDTA-Plasma | Urin  Tid  Kl: | Urin  Volumen  ml |
| --- | --- | --- | --- | --- |
| Baseline  Blod + urin | Kl. 08:25 | + Cystatin C  X | Urin Kl:  08:40 | + 3 tubes urin til Danny |
| Bolus ^51^ Cr  **MR** | Kl. 08:30 |  |  |  |
| Fi T:0 | Kl. 10:05 | X | Start opsamling  Kl. 10:06 |  |
| Fi T:30 | Kl. 10:37 | X | Kl. 10:37 | ml. 12 |
| Fi T:60 | Kl. 11:05 | X | Kl. 11:05 | ml. 46 |
| Fi T:90 | Kl. 11:37 | X | Kl. 11:35 | ml. 175 |
| Iskæmi Start (11:55)  T:0 | Kl. 11:57 | X | Start opsamling  Kl. 11:55 |  |
| T:60 | Kl. 12:56 | X | Kl. 12:55 | ml. 124 |
| Bolus Danegapeptid 10 min før Reperfution T:110 | Kl. 13:45 |  |  |  |
| **ZP1609** T:115 på **is** | Kl 13:50 |  |  |  |
| Iskæmi slut Reperfution  T:120 | Kl. 13:56 | X | Kl. 13:55 | ml. 265 |
| **ZP1609** T:140 på **is** | Kl. 14:15 |  |  |  |
| T:180 + **ZP1609** på **is** | Kl. 14:56 | X | Kl. 14:55 | ml. 105 |
| T: 240 + **ZP1609** på **is**  **MR** | Kl. 15:53 | X | Kl. 15:51 | ml. 175 |
| T:300 + **ZP1609** på **is** eller lige før inf. stop | Kl. 16:55 | + Cystatin C  X | Kl. 16:55 | +3 tubes urin til Danny  ml. 185 |

Plasma til GFR: 3 ml	Urin til GFR: 1 ml
