# Supplementary material for: Testing Danegaptide Effects on Kidney Function after Ischemia/Reperfusion Injury in a New Porcine Two Week Model
Source: PLoS One. 2016 Oct 19;11(10):e0164109. doi: 10.1371/journal.pone.0164109 (PMC5070773; doi:10.1371/journal.pone.0164109)
Supplement: S1 File — (ZIP) [file pone.0164109.s001.zip › PLOS-one/Z13/Z13 B2.doc]

**Gris Z13 B2	Dato: 01.04.14/BS**

T:0 H+V 1 time efter ^51^ Cr bolus

Analyser heparin-plasma: Crea, Carbamid, K, Na, NGAL og TBARS

Analyser Urin: Crea, Carbamid, K, Na, L-FABP, NAG, NGAL og TBARS

| Tid i min |  | Heparin  Plasma  + EDTA  Plasma | Urin  Højre  Tid  Kl: | Urin  Højre  Volumen  ml | Urin  Venstre  Tid  Kl: | Urin  Venstre  Volumen  ml |
| --- | --- | --- | --- | --- | --- | --- |
| Baseline  Blod + urin H+V | Kl. 10:25 | + Cystatin C  X | Urin  Kl. | Ingen baselineurin |  | 3 tubes urin til Danny |
| Bolus ^51^ Cr | Kl. 10:30 |  |  |  |  |  |
| T:0  H+V | Kl. 11:30 | X | H+V  Start ops.  11:35 |  |  | Løber urin ved siden af kateder ? |
| T:30  H+V | Kl. 12:01 | X | H+V  Kl: 12:00 | H+V  **ml: 226** |  |  |
| T:60  H+V | Kl. 12:30 | X | H+V  Kl: 12:30 | H+V  **ml: 130** |  |  |
| T:90  H+V | Kl. 13:00 | X | H+V  Kl: 13.00 | H+V  **ml: 164** |  |  |
| Katedre indsættes |  |  |  |  |  |  |
| T:0 | Kl. 13:25 | X | Start opsamling  Kl. 13:25 |  | Start opsamling  Kl. 13:25 |  |
| T:30 | Kl. 13:53 | X | Kl: 13:55 | **ml: 23** | Kl: 13:55 | **ml: 80** |
| T:60 | Kl. 14:25 | X | Kl: 14:25 | **ml: 12** | Kl: 14:25 | **ml: 60** |
| T:90 | Kl. 15:02 | X | Kl: 15:00 | **ml: 33** | Kl: 15:00 | **ml: 65** |
| T:120  MR | Kl. 15:25 | X | Kl: 15:25 | 3 tubes urin til Danny  **ml: 35** | Kl: 15:25 | 3 tubes urin til Danny  **ml: 136** |

Højre Nyre vægt: 114 g	Plasma til GFR: 3 ml	**Ingen billed af venstre nyre, men makroskopisk ingen forandringer**

Venstre nyre vægt: 111 g	Urin til GFR: 1 ml
