# Supplementary material for: Testing Danegaptide Effects on Kidney Function after Ischemia/Reperfusion Injury in a New Porcine Two Week Model
Source: PLoS One. 2016 Oct 19;11(10):e0164109. doi: 10.1371/journal.pone.0164109 (PMC5070773; doi:10.1371/journal.pone.0164109)
Supplement: S1 File — (ZIP) [file pone.0164109.s001.zip › PLOS-one/Z14/Z14 B2.doc]

**Gris Z14 B2	Dato: 02.04.14/IR**

T:0 H+V 1 time efter ^51^ Cr bolus

Analyser heparin-plasma: Crea, Carbamid, K, Na, NGAL og TBARS

Analyser Urin: Crea, Carbamid, K, Na, L-FABP, NAG, NGAL og TBARS

| Tid i min |  | Heparin  Plasma  + EDTA  Plasma | Urin  Højre  Tid  Kl: | Urin  Højre  Volumen  ml | Urin  Venstre  Tid  Kl: | Urin  Venstre  Volumen  ml |
| --- | --- | --- | --- | --- | --- | --- |
| Baseline  Blod + urin H+V | Kl. 10:12 | + Cystatin C  X | Urin  Kl. 10:20 |  |  | 3 tubes urin til Danny |
| Bolus ^51^ Cr | Kl. 10:15 |  |  |  |  |  |
| T:0  H+V | Kl. 11:17 | X | H+V  Start ops.  11:15 |  |  |  |
| T:30  H+V | Kl. 11:46 | X | H+V  Kl: 11:45 | H+V  **ml: 52** |  |  |
| T:60  H+V | Kl. 12:16 | X | H+V  Kl: 12:15 | H+V  **ml: 48** |  |  |
| T:90  H+V | Kl. 12:46 | X | H+V  Kl: 12:45 | H+V  **ml: 33** |  |  |
| Katedre indsættes |  |  |  |  |  |  |
| T:0 | Kl. 13:11 | X | Start opsamling  Kl. 13:10 |  | Start opsamling  Kl. 13:10 |  |
| T:30 | Kl. 13:41 | X | Kl: 13:40 | **ml: 11** | Kl: 13:40 | **ml: 3+spild**  **- GFR** |
| T:60 | Kl. 14:13 | X | Kl: 14:11 | **ml: 12,5** | Kl: 14:11 | **ml: 7** |
| T:90 | Kl. 14:41 | X | Kl: 14:40 | **ml: 12** | Kl: 14:40 | **ml: 7,5** |
| T:120  MR | Kl. 15:10 | X | Kl: 15:10 | 3 tubes urin til Danny  **ml: 10** | Kl: 15:10 | 3 tubes urin til Danny  **ml: 8** |

Højre Nyre vægt: 112 g	Plasma til GFR: 3 ml	**Foto af delt nyre**

Venstre nyre vægt: 116 g	Urin til GFR: 1 ml
