# Supplementary material for: Testing Danegaptide Effects on Kidney Function after Ischemia/Reperfusion Injury in a New Porcine Two Week Model
Source: PLoS One. 2016 Oct 19;11(10):e0164109. doi: 10.1371/journal.pone.0164109 (PMC5070773; doi:10.1371/journal.pone.0164109)
Supplement: S1 File — (ZIP) [file pone.0164109.s001.zip › PLOS-one/Z16/Z16 B2.doc]

**Gris Z16 B2	Dato: 23.04.14/BS**

T:0 H+V 1 time efter ^51^ Cr bolus

Analyser heparin-plasma: Crea, Carbamid, K, Na, NGAL og TBARS

Analyser Urin: Crea, Carbamid, K, Na, L-FABP, NAG, NGAL og TBARS

| Tid i min |  | Heparin  Plasma  + EDTA  Plasma | Urin  Højre  Tid  Kl: | Urin  Højre  Volumen  ml | Urin  Venstre  Tid  Kl: | Urin  Venstre  Volumen  ml |
| --- | --- | --- | --- | --- | --- | --- |
| Baseline  Blod + urin H+V | Kl. 10.25 | + Cystatin C  X | Urin  Kl. 10.25 |  |  | 3 tubes urin til Danny |
| Bolus ^51^ Cr | Kl. 10.33 |  |  |  |  |  |
| T:0  H+V | Kl. 11.32 | X | H+V  Start ops.  11.33 |  |  |  |
| T:30  H+V | Kl. 12.04 | X | H+V  Kl: 12.05 | H+V  **ml: 205** |  |  |
| T:60  H+V | Kl. 12.33 | X | H+V  Kl: 12.34 | H+V  **ml: 96** |  |  |
| T:90  H+V | Kl. 13.02 | X | H+V  Kl: 13.03 | H+V  **ml: 110** |  |  |
| Katedre indsættes |  |  |  |  |  |  |
| T:0 | Kl. 13.45 | X | Start opsamling  Kl. 13.45 |  | Start opsamling  Kl. 13.45 |  |
| T:30 | Kl. 14.15 | X | Kl: 14.16 | **ml: 9** | Kl: 14.16 | **ml: 24** |
| T:60 | Kl. 14.45 | X | Kl: 14.45 | **ml: 9** | Kl: 14.45 | **ml: 23** |
| T:90 | Kl. 15.15 | X | Kl: 15.15 | **ml: 7** | Kl: 15.15 | **ml: 19** |
| T:120  MR | Kl. 15.43 | X | Kl: 15.43 | 3 tubes urin til Danny  **ml: 6** | Kl: 15.43 | 3 tubes urin til Danny  **ml: 13** |

Højre Nyre vægt: 124 g	Plasma til GFR: 3 ml	**Foto af delt nyre**

Venstre nyre vægt: 119 g	Urin til GFR: 1 ml
