# Supplementary material for: Testing Danegaptide Effects on Kidney Function after Ischemia/Reperfusion Injury in a New Porcine Two Week Model
Source: PLoS One. 2016 Oct 19;11(10):e0164109. doi: 10.1371/journal.pone.0164109 (PMC5070773; doi:10.1371/journal.pone.0164109)
Supplement: S1 File — (ZIP) [file pone.0164109.s001.zip › PLOS-one/Z18/Z18 B1 med ZP1609.doc]

**Gris Z18 B1**	Dato: 10.04.14/BS

Fi T:0 1 time efter ^51^ Cr bolus.

ZP1609	T:115, T:140, T:180, T:240 og T:300 lige før infutionsstop. 1 ml EDTA-P

Analyser heparin-plasma: Crea, Carbamid, K, Na, NGAL, Cystatin C og TBARS

Analyser Urin: Crea, Carbamid, K, Na, L-FABP, NAG, NGAL og TBARS

| Tid i min |  | Heparin  Plasma  + EDTA-Plasma | Urin  Tid  Kl: | Urin  Volumen  ml |
| --- | --- | --- | --- | --- |
| Baseline  Blod + urin | Kl. 08.45 | + Cystatin C  X | Urin Kl:  08.55 | + 3 tubes urin til Danny |
| Bolus ^51^ Cr  **MR** | Kl. 08.50 |  |  |  |
| Fi T:0 | Kl. 10.24 | X | Start opsamling  Kl. 10.24 |  |
| Fi T:30 | Kl. 10.54 | X | Kl. 10.54 | ml. 12 |
| Fi T:60 | Kl. 11.25 | X | Kl. 11.24 | ml. 9 |
| Fi T:90 | Kl. 11.54 | X | Kl. 11.55 | ml. 18 |
| Iskæmi Start  T:0 | Kl. 12.11 | X | Start opsamling  Kl. 12.11 |  |
| T:60 | Kl. 13.11 | X | Kl. 13.11 | ml. 22 |
| Bolus Danegapeptid 10 min før Reperfution T:110 | Kl. 14.01 |  |  |  |
| **ZP1609** T:115 på **is** | Kl 14.06 |  |  |  |
| Iskæmi slut Reperfution  T:120 | Kl. 14.11 | X | Kl. 14.12 | ml. 21 |
| **ZP1609** T:140 på **is** | Kl. 14.31 |  |  |  |
| T:180 + **ZP1609** på **is** | Kl. 15.11 | X | Kl. 15.11 | ml. 14 |
| T: 240 + **ZP1609** på **is**  **MR** | Kl. 16.06 | X | Urinopsaml.  **15.45-16.08**  Kl. 16.08 | ml. 30 |
| T:300 + **ZP1609** på **is** | Kl. 16.11 | + Cystatin C  X | Kl. 16.12 | +3 tubes urin til Danny  ml. 228 |

Plasma til GFR: 3 ml	Urin til GFR: 1 ml
