# Supplementary material for: Testing Danegaptide Effects on Kidney Function after Ischemia/Reperfusion Injury in a New Porcine Two Week Model
Source: PLoS One. 2016 Oct 19;11(10):e0164109. doi: 10.1371/journal.pone.0164109 (PMC5070773; doi:10.1371/journal.pone.0164109)
Supplement: S1 File — (ZIP) [file pone.0164109.s001.zip › PLOS-one/Z21/Z21 B1 med ZP1609.doc]

**Gris Z21 B1**	Dato: 05.06.14/BS

Fi T:0 1 time efter ^51^ Cr bolus.

ZP1609	T:115, T:140, T:180, T:240 og T:300 lige før infutionsstop. 1 ml EDTA-P

Analyser heparin-plasma: Crea, Carbamid, K, Na, NGAL, Cystatin C og TBARS

Analyser Urin: Crea, Carbamid, K, Na, L-FABP, NAG, NGAL og TBARS

| Tid i min |  | Heparin  Plasma  + EDTA-Plasma | Urin  Tid  Kl: | Urin  Volumen  ml |
| --- | --- | --- | --- | --- |
| Baseline  Blod + urin | Kl. 08.35 | + Cystatin C  X | Urin Kl:  08.45 | + 3 tubes urin til Danny**(Mangler)** |
| Bolus ^51^ Cr  **MR** | Kl. 08.50 |  |  |  |
| Fi T:0 | Kl. 10.25 | X | Start opsamling  Kl. 10.35 |  |
| Fi T:30 | Kl. 10.55 | X | Kl. 10.56 | ml. 7 |
| Fi T:60 | Kl. 11.25 | X | Kl. 11.26 | ml. 17 |
| Fi T:90 | Kl. 11.57 | X | Kl. 11.58 | ml. 18 |
| Iskæmi Start  T:0 | Kl. 12.13 | X | Start opsamling  Kl. 12.13 |  |
| T:60 | Kl. 13.13 | X | Kl. 13.13 | ml. 89 |
| Bolus Danegapeptid 10 min før Reperfution T:110 | Kl. 14.03 |  |  |  |
| **ZP1609** T:115 på **is** | Kl 14.08 |  |  |  |
| Iskæmi slut Reperfution  T:120 | Kl. 14.13 | X | Kl. 14.14 | ml. 40 |
| **ZP1609** T:140 på **is** | Kl. 14.33 |  |  |  |
| T:180 + **ZP1609** på **is** | Kl. 15.13 | X | 1) Kl. 14.14-14.43  2) 14.43-15.13 | 1) 85 ml  2) 13 ml  * |
| T: 240 + **ZP1609** på **is**  **MR** | - blod  Kl. 16.13 | X | Kl. 16.20 | ml. 66 |
| T:300 + **ZP1609** på **is** eller lige før inf. stop | Kl. 16.11 | + Cystatin C  X | Kl. 16.11 | +3 tubes urin til Danny  ml. 295 |

Plasma til GFR: 3 ml	Urin til GFR: 1 ml

*GFR på begge tider. Urin til fryser blanding af begge portioner.
