# Supplementary material for: Testing Danegaptide Effects on Kidney Function after Ischemia/Reperfusion Injury in a New Porcine Two Week Model
Source: PLoS One. 2016 Oct 19;11(10):e0164109. doi: 10.1371/journal.pone.0164109 (PMC5070773; doi:10.1371/journal.pone.0164109)
Supplement: S1 File — (ZIP) [file pone.0164109.s001.zip › PLOS-one/Z22/Z22 B1 med ZP1609.doc]

**Gris Z22 B1**	Dato: 05.06.14/KC

Fi T:0 1 time efter ^51^ Cr bolus.

ZP1609	T:115, T:140, T:180, T:240 og T:300 lige før infutionsstop. 1 ml EDTA-P

Analyser heparin-plasma: Crea, Carbamid, K, Na, NGAL, Cystatin C og TBARS

Analyser Urin: Crea, Carbamid, K, Na, L-FABP, NAG, NGAL og TBARS

| Tid i min |  | Heparin  Plasma  + EDTA-Plasma | Urin  Tid  Kl: | Urin  Volumen  ml |
| --- | --- | --- | --- | --- |
| Baseline  Blod + urin | Kl. 08.20 | + Cystatin C  X | Urin Kl:  08.30 | + 3 tubes urin til Danny |
| Bolus ^51^ Cr  **MR** | Kl. 08.30 |  |  |  |
| Fi T:0 | Kl. 09.55 | X | Start opsamling  Kl. 09.56 |  |
| Fi T:30 | Kl. 10.25 | X | Kl. 10.25 | ml. 270 |
| Fi T:60 | Kl. 10.55 | X | Kl. 10.55 | ml. 310 |
| Fi T:90 | Kl. 11.25 | X | Kl. 11.25 | ml. 175 |
| Iskæmi Start  T:0 | Kl. 11.44 | X | Start opsamling  Kl. 11.44 |  |
| T:60 | Kl. 12.44 | X | Kl. 12.44 | ml. 180 |
| Bolus Danegapeptid 10 min før Reperfution T:110 | Kl. 13.34 |  |  |  |
| **ZP1609** T:115 på **is** | Kl 13,39 |  |  |  |
| Iskæmi slut Reperfution  T:120 | Kl. 13.44 | X | Kl. 13.44 | ml. 320 |
| **ZP1609** T:140 på **is** | Kl. 14.04 |  |  |  |
| T:180 + **ZP1609** på **is** | Kl. 14.44 | X | Kl. 14.44 | ml. 35 |
| T: 240 + **ZP1609** på **is**  **MR** | Kl. 15.46 | X | Kl. 15.46 | ml. 210 |
| T:300 + **ZP1609** på **is** eller lige før inf. stop | Kl. 16.44 | + Cystatin C  X | Kl. 16.44 | +3 tubes urin til Danny  ml. 315 |

Plasma til GFR: 3 ml	Urin til GFR: 1 ml
