# Supplementary material for: Testing Danegaptide Effects on Kidney Function after Ischemia/Reperfusion Injury in a New Porcine Two Week Model
Source: PLoS One. 2016 Oct 19;11(10):e0164109. doi: 10.1371/journal.pone.0164109 (PMC5070773; doi:10.1371/journal.pone.0164109)
Supplement: S1 File — (ZIP) [file pone.0164109.s001.zip › PLOS-one/Z24/Z24 B2.doc]

**Gris Z24 B2	Dato: 26.06.14/BKS**

T:0 H+V 1 time efter ^51^ Cr bolus

Analyser heparin-plasma: Crea, Carbamid, K, Na, NGAL og TBARS

Analyser Urin: Crea, Carbamid, K, Na, L-FABP, NAG, NGAL og TBARS

| Tid i min |  | Heparin  Plasma  + EDTA  Plasma | Urin  Højre  Tid  Kl: | Urin  Højre  Volumen  ml | Urin  Venstre  Tid  Kl: | Urin  Venstre  Volumen  ml |
| --- | --- | --- | --- | --- | --- | --- |
| Baseline  Blod + urin H+V | Kl. 08.40 | + Cystatin C  X | Urin  Kl. 08.40 |  |  | 3 tubes urin til Danny |
| Bolus ^51^ Cr | Kl. 08.45 |  |  |  |  |  |
| T:0  H+V | Kl. 10.31 | X | H+V  Start ops.  10.33 |  |  |  |
| T:30  H+V | Kl. 11.01 | X | H+V  Kl: 11.01 | H+V  **ml: 3** | Fryse-prøver 1 rør |  |
| T:60  H+V | Kl. 11.31 | X | H+V  Kl: 11.32 | H+V  **ml: 13** |  |  |
| T:90  H+V | Kl. 12.01 | X | H+V  Kl: 12.02 | H+V  **ml: 8** |  |  |
| Katedre indsættes |  |  |  |  |  |  |
| T:0 | Kl. 12.26 | X | Start opsamling  Kl. 12.26 |  | Start opsamling  Kl. 12.26 |  |
| T:30 | Kl. 12.56 | X | Kl: 12.57 | **ml: 8** | Kl: 12.57 | **ml: 8** |
| T:60 | Kl. 13.26 | X | Kl: 13.26 | **ml: 7** | Kl: 13.26 | **ml: 7** |
| T:90 | Kl. 13.56 | X | Kl: 13.56 | **ml: 7** | Kl: 13.56 | **ml: 7** |
| T:120  MR | Kl. 14.26 | X | Kl: 14.26 | 3 tubes urin til Danny  **ml: 8** | Kl: 14.26 | 3 tubes urin til Danny  **ml: 7** |

Højre Nyre vægt: 111,36 g	Plasma til GFR: 3 ml	**Foto af delt nyre**

Venstre nyre vægt: 90.40 g	Urin til GFR: 1 ml
