# Supplementary material for: Testing Danegaptide Effects on Kidney Function after Ischemia/Reperfusion Injury in a New Porcine Two Week Model
Source: PLoS One. 2016 Oct 19;11(10):e0164109. doi: 10.1371/journal.pone.0164109 (PMC5070773; doi:10.1371/journal.pone.0164109)
Supplement: S1 File — (ZIP) [file pone.0164109.s001.zip › PLOS-one/Z3/Z3 B1.doc]

**Gris Z3 B1**

Fi T:0 1 time efter ^51^ Cr bolus.

Analyser heparin-plasma: Crea, Carbamid, K, Na, NGAL og TBARS

Analyser Urin: Crea, Carbamid, K, Na, L-FABP, NAG, NGAL og TBARS

Dato: 16.01.14

| Tid i min |  | Heparin  Plasma  + EDTA  Plasma | Urin  Tid  Kl: | Urin  Volumen  ml |
| --- | --- | --- | --- | --- |
| Baseline  Blod + urin | Kl. 08:20 | X | Urin Kl.  09:30 |  |
| Bolus ^51^ Cr  **MR** | Kl. 08:25 |  |  |  |
| Fi T:0  Veneblod | Kl. 09:35 | X | Start opsamling  Kl. 09:37 |  |
| Fi T:30 | Kl. 10:15 | X | Kl. 10:20 | **0,5 ml til GFR**  ml. 2,5 |
| Fi T:60 | Kl. 10:47 | X | Kl. 10:50 | **0,5 ml til GFR**  ml. 2,5 |
| Fi T:90 | Kl. 11:16 | X | Kl. 11:17 | ml. 14 |
| Iskæmi Start  T:0 | Kl. 11:50 | X | Start opsamling  Kl. 11.50 |  |
| T:60 | Kl. 12:52 | X | Kl. 12:50 | ml. 12,5 |
| Bolus Danegapeptid 10 min før Reperfution | Kl. 13:42 |  |  |  |
| Iskæmi slut Reperfution  T:120 | Kl. 13:50 | X | Kl. 13:49 | ml. 17 |
| T:180 | Kl. 14:50 | X | Kl. 14:50 | ml. 35 |
| T: 240  **MR** | Kl. 15:50 | X | Kl. 15:50 | ml. 52 |
| T:300 | Kl. 16:50 | X | Kl. 16:50 | ml. 84,5 |

Plasma til GFR: 3 ml	Urin til GFR: 1 ml
