# Supplementary material for: Testing Danegaptide Effects on Kidney Function after Ischemia/Reperfusion Injury in a New Porcine Two Week Model
Source: PLoS One. 2016 Oct 19;11(10):e0164109. doi: 10.1371/journal.pone.0164109 (PMC5070773; doi:10.1371/journal.pone.0164109)
Supplement: S1 File — (ZIP) [file pone.0164109.s001.zip › PLOS-one/Z3/Z3 B2.doc]

**Gris Z3 B2**

T:0 H+V 1 time efter ^51^ Cr bolus

Analyser heparin-plasma: Crea, Carbamid, K, Na, NGAL og TBARS

Analyser Urin: Crea, Carbamid, K, Na, L-FABP, NAG, NGAL og TBARS

Dato: 30.01.14	**Kl. 11:40: Cr 51 ikke løbet ind de første 50 min.**

| Tid i min | Kl. | Heparin  Plasma  + EDTA  Plasma | Urin  Højre  Tid  Kl: | Urin  Højre  Volumen  ml | Urin  Venstre  Tid  Kl: | Urin  Venstre  Volumen  ml |
| --- | --- | --- | --- | --- | --- | --- |
| Baseline  Blod + urin H+V | Kl. 10:40 | X | Urin  Kl. 11:20 |  |  | **Baseline GFR 2 ml** |
| Bolus ^51^ Cr | Kl. 10:50 |  |  |  |  |  |
| T:0  H+V | Kl. 11:55 | X | H+V  Start ops. 11:50 |  |  |  |
| T:30  H+V | Kl. 12:20 | X | H+V  Kl: 12:20 | H+V  **ml: 8** |  |  |
| T:60  H+V | Kl. 12:50 | X | H+V  Kl: 12:50 | H+V  **ml: 11** |  |  |
| T:90  H+V | Kl. 13:20 | X | H+V  Kl: 13:20 | H+V  **ml: 8** |  |  |
| Katedre indsættes |  |  |  |  |  |  |
| T:0 | Kl. 13:50 | X | Start opsamling  Kl. 13:49 |  | Start opsamling  Kl. 13:49 |  |
| T:30 | Kl. 14:20 | X | Kl: 14:20 | **ml: 7** | Kl: 14:20 | **ml: 8,5** |
| T:60 | Kl. 14:50 | X | Kl: 14:49 | **ml: 6**  **Spild efter 8 min** | Kl: 14:49 | **ml: 14** |
| T:90 | Kl. 15:28 | X | Kl: 15:25 | **ml: 14** | Kl: 15:25 | **ml: 19** |
| T:120  MR | Kl. 15:55 | X | Kl: 15:55 | **ml: 8** | Kl: 15:55 | **ml: 20** |

Højre Nyre vægt: 94,30	Plasma til GFR: 3 ml	**Foto af delt nyre**

Venstre nyre vægt: 89,63	Urin til GFR: 1 ml
