# Supplementary material for: Testing Danegaptide Effects on Kidney Function after Ischemia/Reperfusion Injury in a New Porcine Two Week Model
Source: PLoS One. 2016 Oct 19;11(10):e0164109. doi: 10.1371/journal.pone.0164109 (PMC5070773; doi:10.1371/journal.pone.0164109)
Supplement: S1 File — (ZIP) [file pone.0164109.s001.zip › PLOS-one/Z5/Z5 B1 med ZP1609.doc]

**Gris Z5 B1**	Dato: 12.02.14

Fi T:0 1 time efter ^51^ Cr bolus. BS

ZP1609	T:115, T:140, T:180, T:240 og T:300 lige før infutionsstop. 1 ml EDTA-P

Analyser heparin-plasma: Crea, Carbamid, K, Na, NGAL og TBARS

Analyser Urin: Crea, Carbamid, K, Na, L-FABP, NAG, NGAL og TBARS

| Tid i min |  | Heparin  Plasma  + EDTA-Plasma | Urin  Tid  Kl: | Urin  Volumen  ml |
| --- | --- | --- | --- | --- |
| Baseline  Blod + urin | Kl. 08:15 | X | Urin Kl:  08:15 |  |
| Bolus ^51^ Cr  **MR** | Kl. 08:20 |  |  | - ^51^Cr fra bolus til 09:45  5 ml bolus kl. 09:45 |
| Fi T:0 | Kl. 09:55 | X | Start opsamling  Kl. 09:55 |  |
| Fi T:30 | Kl. 10:25 | X | Kl. 10:25 | ml. 7 |
| Fi T:60 | Kl. 10:55 | X | Kl. 10:55 | ml. 12 |
| Fi T:90 | Kl. 11:25 | X | Kl. 11:25 | ml. 30 |
| Iskæmi Start  T:0 | Kl. 12:10 | X | Start opsamling  Kl. 12:10 |  |
| T:60 | Kl. 13:10 | X | Kl. 13:10 | ml. 100 |
| Bolus Danegapeptid 10 min før Reperfution T:110 | Kl. 14:00 |  |  |  |
| **ZP1609** T:115 på **is** | Kl 14:05 |  |  |  |
| Iskæmi slut Reperfution  T:120 | Kl. 14:10 | X | Kl. 14:10 | ml. 130 |
| **ZP1609** T:140 på **is** | Kl. 14:30 |  |  |  |
| T:180 + **ZP1609** på **is** | Kl. 15:10 | X | Kl. 15:10 | ml. 40 |
| T: 240 + **ZP1609** på **is**  **MR** | Veneblod fra Øre  Kl. 16:20 | X | Kl. 16:10 | ml. 110 |
| T:300 + **ZP1609** på **is**  **GFR 1,5 ml** | Kl. 17:10 | X | Kl. 17:10 | 1. 80 ml kl.16:10-16:30  2. 80 ml kl. 16:50-17:10 |

Plasma til GFR: 3 ml	Urin til GFR: 1 ml
