# Supplementary material for: Testing Danegaptide Effects on Kidney Function after Ischemia/Reperfusion Injury in a New Porcine Two Week Model
Source: PLoS One. 2016 Oct 19;11(10):e0164109. doi: 10.1371/journal.pone.0164109 (PMC5070773; doi:10.1371/journal.pone.0164109)
Supplement: S1 File — (ZIP) [file pone.0164109.s001.zip › PLOS-one/Z6/Z6 B2.doc]

**Gris Z6 B2**

T:0 H+V 1 time efter ^51^ Cr bolus	/ KC

Analyser heparin-plasma: Crea, Carbamid, K, Na, NGAL og TBARS

Analyser Urin: Crea, Carbamid, K, Na, L-FABP, NAG, NGAL og TBARS

Dato: 27.02.14

| Tid i min |  | Heparin  Plasma  + EDTA  Plasma | Urin  Højre  Tid  Kl: | Urin  Højre  Volumen  ml | Urin  Venstre  Tid  Kl: | Urin  Venstre  Volumen  ml |
| --- | --- | --- | --- | --- | --- | --- |
| Baseline  Blod + urin H+V | Kl. 10:15 | + Cystatin C  X | Urin  Kl. | **Ingen baseline urin** |  | 1 tubes urin til Danny T:30 H+V |
| Bolus ^51^ Cr | Kl. 10:20 |  |  |  |  |  |
| T:0  H+V | Kl. 11:20 | X | H+V  Start ops.  11:20 |  |  |  |
| T:30  H+V | Kl. 11:51 | X | H+V  Kl: 11:50 | H+V  **ml: 2,5** | **0,5 ml til GFR**  - ekstra pr. |  |
| T:60  H+V | Kl. 12:21 | X | H+V  Kl: 12:21 | H+V  **ml: 8** |  |  |
| T:90  H+V | Kl. 12:50 | X | H+V  Kl: 12:50 | H+V  **ml: 9** |  |  |
| Katedre indsættes |  |  |  |  |  |  |
| T:0 | Kl. 13:15 | X | Start opsamling  Kl. 13:15 |  | Start opsamling  Kl. 13:15 |  |
| T:30 | Kl. 13:45 | X | Kl: 13:45 | **ml: 14** | Kl: 13:45 | **ml: 20** |
| T:60 | Kl. 14:15 | X | Kl: 14:15 | **ml: 9** | Kl: 14:15 | **ml: 12,5** |
| T:90 | Kl. 14:45 | X | Kl: 14:45 | **ml: 14** | Kl: 14:45 | **ml: 7,5** |
| T:120  MR | Kl. 15:19 | X | Kl: 15:18 | 3 tubes urin til Danny  **ml: 10** | Kl: 15:18 | 3 tubes urin til Danny  **ml: 30** |

Højre Nyre vægt:	Plasma til GFR: 3 ml	**Foto af delt nyre**

Venstre nyre vægt:	Urin til GFR: 1 ml
